# Psychological Factors and Personality Traits Influencing the Experience and Perception of Pain in Women Undergoing Mammography: Protocol for a Cross-Sectional Study

**DOI:** 10.2196/92804

**Published:** 2026-08-25

**Authors:** Irene Neophytou, George Charalambous, Eleni Jelastopulu

**Affiliations:** 1 Department of Nursing, Frederick University, Gianni Freiderikou 7, Nicosia, Nicosia, 1036, Cyprus, 357 22394394; 2 Laboratory of Hygiene, Epidemiology and Quality of Life, School of Medicine, University of Patras, Patras, West Greece, Greece

**Keywords:** screening mammography, pain catastrophizing, depression, anxiety, pain

## Abstract

**Background:**

Breast cancer screening through mammography is essential for early detection and improved survival; however, pain and discomfort during the procedure remain major barriers to women’s participation. Pain perception during mammography is influenced not only by technical factors but also by psychological variables such as anxiety, depression, pain catastrophizing, and personality traits. Despite growing evidence, findings on the role of psychological factors remain inconsistent, and the contribution of personality traits remains largely unexplored, highlighting the need for further investigation.

**Objective:**

This study aims to investigate associations among psychological factors (anxiety, depression, and pain catastrophizing), Ten-Item Personality Inventory personality traits, and pain perception and experience in women undergoing routine mammography at public and private radiology centers in Cyprus. The study hypothesizes that higher anxiety levels and first-time mammography experience are associated with greater perceived pain during mammography.

**Methods:**

This cross-sectional study will include approximately 380 women aged 35 to 75 years undergoing routine screening mammography at public and private radiology centers in Cyprus. Participants will complete the Patient Health Questionnaire-4, the Pain Catastrophizing Scale, the Ten-Item Personality Inventory, and a demographic and clinical questionnaire before the examination. The primary outcome will be perceived pain intensity during mammography, assessed immediately after the examination using a Visual Analog Scale. Psychological factors, including anxiety, depression, pain catastrophizing, and personality traits, will be evaluated as the main independent variables. Secondary analyses will examine the role of demographic and clinical characteristics, as well as previous mammography experience, in pain perception. Descriptive statistics will summarize participant characteristics, and associations between study variables and pain intensity will be examined using Pearson or Spearman correlation analyses and hierarchical multiple linear regression models adjusted for relevant confounders. The study will be conducted and reported in accordance with the STROBE (Strengthening the Reporting of Observational Studies in Epidemiology) guidelines for observational studies.

**Results:**

Participant recruitment and data collection began in March 2026. By June 2026, 50 women had been enrolled in the study. Recruitment and data collection are expected to continue through September 2026. Data analysis is anticipated to be completed by December 2026, with dissemination of findings and manuscript submission planned for 2027. Following study initiation, the study setting was expanded to include a public radiology center to facilitate recruitment and enhance sample representativeness.

**Conclusions:**

If the study findings support the proposed associations, early identification and management of psychological factors related to pain perception during mammography may improve women’s overall experience, enhance comfort during the procedure, and potentially increase adherence to breast cancer screening programs.

## Introduction

### Background

Breast cancer is the most common cancer among women worldwide, with about 1 in 8 women diagnosed during their lifetime [1]. Early detection through screening mammography significantly improves prognosis and survival outcomes, with 5-year survival rates reaching up to 99% for early-stage breast cancer [1]. Despite the well-documented benefits of screening, many eligible women avoid or delay participation in mammography programs [2].

One major reason women hesitate to participate in mammography screening is the pain and discomfort they experience during the procedure. Pain is not just a temporary annoyance; it significantly influences health behaviors, as women who experience severe pain are less likely to return for future screenings [3-5].

Pain perception and pain experience are not determined solely by biological or technical factors but are significantly influenced by psychological variables [6]. Factors such as anxiety, pain catastrophizing, fear, and previous pain experiences have been linked to increased pain perception across various clinical and diagnostic settings [7-9].

According to the biopsychosocial model of pain, pain perception is influenced by the dynamic interaction of physiological, psychological, and social factors. As pain is a subjective experience, it is influenced by a broad range of cognitive, behavioral, and affective processes, through which psychological factors and personality traits may contribute to individual differences in pain perception [10].

Despite growing research interest in mammography-related pain, the evidence remains fragmented and inconclusive. Although anxiety, depressive symptoms, and pain catastrophizing have repeatedly been identified as potential predictors of pain during mammography, findings remain inconsistent, making it unclear which psychological variables are most strongly associated with pain perception and whether these factors exert independent effects when examined simultaneously [11-18].

Anxiety has been the most extensively studied psychological factor, with most studies reporting a positive association between higher anxiety levels and greater pain during mammography, although some have found no statistically significant relationship [11-15].

Likewise, evidence regarding depressive symptoms and pain catastrophizing remains inconsistent, with studies reporting both significant and nonsignificant associations with pain intensity [14-18].

These discrepancies may stem from differences in study design, population characteristics, and variability in how variables are selected and assessed, highlighting the need for further research using comprehensive models that examine multiple psychological factors concurrently.

In addition to psychological factors, personality traits may also influence how individuals perceive and respond to painful experiences. However, the role of personality traits in mammography-related pain remains largely unexplored. To date, only one study has specifically investigated the association between personality characteristics and pain experienced during mammography.

Montoro et al [14] reported moderate correlations between extraversion, psychoticism, and pain intensity, and a weak correlation with neuroticism; however, none of these associations reached statistical significance. Therefore, the potential contribution of personality traits to mammography-related pain remains unclear and warrants further investigation.

Importantly, no previous study has comprehensively examined anxiety, depressive symptoms, pain catastrophizing, and personality traits within the same analytical framework. As a result, it remains unclear whether personality characteristics contribute uniquely to pain perception beyond established psychological factors. Addressing this gap may provide a more comprehensive understanding of the psychological determinants of mammography-related pain and help identify women at greater risk of experiencing pain during screening.

Therefore, the current literature is characterized by three important limitations: (1) inconsistent findings on the associations between anxiety, depression, and pain catastrophizing and mammography-related pain; (2) extremely limited evidence on the role of personality traits, with only one study investigating this relationship; and (3) the absence of studies that simultaneously examine these psychological and personality-related factors within the same population.

To address these evidence gaps, this study builds upon our previously published work on this topic. A protocol for a systematic review investigating psychological factors that influence pain perception and pain experience during mammography was first published in *JMIR Research Protocols* [19] and was followed by the completed systematic review [20]. The systematic review demonstrated that anxiety was the psychological factor most consistently associated with mammography-related pain, whereas evidence on depressive symptoms, pain catastrophizing, and personality traits remained inconclusive. It also identified important evidence gaps, particularly a lack of studies simultaneously examining multiple psychological and personality-related factors within the same analytical framework. These findings informed the design of this study and the selection of anxiety, depressive symptoms, pain catastrophizing, and personality traits as the key psychological predictors examined in relation to mammography-related pain. Building upon the systematic review, this cross-sectional study investigates these factors concurrently within a single population to determine their independent associations with pain perception and pain experience during mammography.

For the purposes of this study, pain perception refers to the intensity of pain reported by participants immediately after mammography using the Visual Analog Scale (VAS), whereas pain experience encompasses the broader subjective experience of mammography, including participants’ reflections captured through the open-ended questions.

### Objectives

The aim of this study is to explore the influence of psychological factors and personality traits on pain perception and experience during mammography. Specifically, it analyzes variations in pain scores during routine mammographic screenings at 2 radiology centers in Cyprus and examines how psychological variables—such as anxiety, depression, pain catastrophizing, and personality traits—affect the pain experienced during the procedure.

The specific objectives of the study are as follows:

- To assess the level of pain experienced by women during mammography.
- To investigate the relationship between anxiety levels and the intensity of perceived pain during mammography.
- To investigate the role of depression in pain intensity during mammography.
- To assess whether pain catastrophizing is linked to the pain experienced during mammography.
- To examine the relationship between personality traits and pain perception and experience during mammography.
- To explore whether previous mammography experience (first-time vs repeat examinations) moderates the relationship between anxiety and perceived pain during screening mammography.

Given the inconsistent or limited evidence in the existing literature, depression, pain catastrophizing, and personality traits will be examined without prespecified directional hypotheses. The study is based on the following research hypotheses:

- H1: Higher levels of anxiety will be positively associated with increased perceived pain during mammography.
- H2: Women undergoing mammograms for the first time are expected to report higher perceived pain levels than women with previous mammography experience.

## Methods

### Study Design

This study uses a cross-sectional design. Data are mainly collected through a quantitative approach using validated self-report psychometric questionnaires. Additionally, open-ended questions are included to allow participants to describe their mammography experience in their own words, providing interpretive support for the quantitative results.

Data collection takes place under real clinical conditions among women undergoing routine screening mammography. The study protocol adheres to the STROBE (Strengthening the Reporting of Observational Studies in Epidemiology) reporting guidelines for observational studies (Checklist 1).

Including both public and private radiology centers was intended to enhance sample diversity and improve the representativeness of the sample of women undergoing routine screening mammography in Cyprus. The cross-sectional design was chosen because it allows efficient investigation of associations between psychological factors, personality traits, and pain perception during mammography in real-world clinical settings. Although this design can identify potential associations and generate hypotheses for future research, it does not permit causal conclusions. The findings are expected to help to address gaps and inconsistencies in the existing literature on the psychological determinants of mammography-related pain, particularly in Mediterranean populations, where relevant evidence remains limited.

### Setting

The study is conducted in the Radiology Department at the Nicosia Polyclinic, a private hospital in the Nicosia District of Cyprus, and in a public radiology center in Cyprus.

Data were collected in 2 stages:

- Before undergoing mammography, participants complete the psychometric questionnaires.
- Immediately after mammography, pain levels are assessed, and additional questions about the examination experience are answered.

### Participants

The study includes healthy women who meet all of the following inclusion criteria:

- Age between 35 and 75 years.
- Attendance at the Radiology Department’s Mammography Unit at the Nicosia Polyclinic, a public mammography center, was for routine screening mammography.
- Ability to understand, read, and write Greek to ensure reliable completion of the study questionnaires.
- Women of all employment statuses, professions, and social roles will be eligible to participate.
- Participation was voluntary, with written informed consent provided after a full explanation of the study’s aims, procedures, and the nature of the research.

Women exhibiting one or more of the following traits are excluded from the study:

- Neurological disorders such as dementia, Alzheimer disease, and traumatic brain injury.
- Serious physical illnesses, such as active cancer, including breast cancer.
- Clinical signs of breast disease (eg, suspicion of a palpable breast mass).
- Presence of breast implants.
- History of a diagnosed severe psychiatric disorder (eg, psychotic disorders, schizophrenia) that could influence pain perception or the ability to provide reliable self-reports.
- Pregnancy or breastfeeding should be considered a contraindication to mammography due to exposure to ionizing radiation.
- Difficulty understanding and completing questionnaires in Greek.

Participants are recruited using consecutive sampling. Women who meet the inclusion criteria are approached sequentially in the Mammography Unit waiting area upon their arrival for a scheduled routine screening examination. The use of consecutive sampling is justified by the easy access to the target population and the practicality of data collection in real clinical settings. This method allows the inclusion of women undergoing the mammography procedure in real time, thereby increasing the ecological validity and reliability of the study results.

### Variables

To examine the independent associations between psychological factors, broad personality dimensions, and the primary outcome (perceived pain intensity), hierarchical multiple linear regression analysis will be conducted. The main independent variables include psychological factors—specifically anxiety, depression, and pain catastrophizing—as well as personality traits. Potential confounding factors that may influence the relationship between psychological factors and pain perception include age, previous mammography experience, breast size, menopausal status, phase of the menstrual cycle, smoking status, presence of chronic illness, and educational level. The study will also explore whether previous mammography experience (first-time vs repeat examination) moderates the relationship between psychological factors—especially anxiety—and pain perception. Based on previous literature, the study will examine whether the association between anxiety and perceived pain differs between women undergoing their first mammogram and those with previous mammography experience.

Other factors, such as analgesic use, breast compression force, and breast density, have also been reported as potential confounders of pain perception. However, these variables are not assessed in this study because it relies exclusively on participant-completed questionnaires and does not involve the collection of clinical or imaging data.

Since the study involves healthy women undergoing screening mammography, no medical diagnostic criteria are used. All participants are asymptomatic, show no signs of breast disease, and have no history of breast cancer.

### Data Sources or Measurement

Data are gathered from primary sources through paper-based self-report questionnaires completed by participants before and after the mammography examination, under the supervision of the principal investigator. All measurements are taken under standardized conditions (the same setting, duration, and investigator) to ensure data reliability and comparability. Data are gathered directly from participants using the instruments described in the following sections.

#### Demographic and Clinical Information Form

A structured demographic and clinical questionnaire (Multimedia Appendix 1) is used to gather information on age, educational level (according to the Greek or Cypriot educational system), marital status, whether the participants have children, employment status, living arrangements (living alone or with others), economic sector, place of residence, self-reported health status, psychological well-being, smoking status, presence of chronic diseases, neurological disorders, serious physical illnesses, history of psychiatric disorders, presence of breast implants, menopausal status (premenopausal or postmenopausal), and the phase of the menstrual cycle (when applicable). Additionally, mammography-related details are recorded, including prior mammography history (first-time vs repeat examination), the year of the most recent mammogram, and any prior pain experienced during mammography. Breast size is estimated based on self-reported bra size.

#### Psychometric Questionnaires

Anxiety and depressive symptoms are assessed using the Patient Health Questionnaire-4 (PHQ-4), a brief, valid, and reliable screening tool with 4 items [21]. The questionnaire consists of 2 items for anxiety and 2 for depressive symptoms [22]. The PHQ-4 has been translated, culturally adapted, and validated in Greek [23]. Written permission for its use in this study has been obtained from the original authors. The PHQ-4 was selected for its brief and efficient assessment of anxiety and depressive symptoms while minimizing participant burden. Its brevity makes it particularly suitable for clinical settings where participants must complete multiple questionnaires. Furthermore, the Greek version of the PHQ-4 has demonstrated good psychometric properties, including acceptable internal consistency (Cronbach α=0.80), excellent test-retest reliability (intraclass correlation=0.96), and satisfactory validity in the Greek general population [23].

Personality traits are evaluated using the Ten-Item Personality Inventory (TIPI), a short self-report questionnaire with 10 items [24]. The TIPI assesses 5 main personality traits: extraversion, agreeableness, conscientiousness, emotional stability, and openness to experience. A Greek version of the TIPI is available from the official source of the instrument’s developers [25]. The TIPI was selected because it offers a brief assessment of the 5 major personality dimensions and requires minimal time to complete. This was considered particularly important, given that participants must complete several questionnaires before undergoing mammography. The instrument was developed for research settings in which more comprehensive personality measures may be impractical due to time constraints. Although the TIPI has lower internal consistency than more comprehensive personality inventories, because each personality dimension is assessed with only 2 items, it has demonstrated acceptable validity and is considered appropriate for research in time-constrained clinical settings.

Pain catastrophizing is evaluated using the Pain Catastrophizing Scale (PCS), which includes 13 statements describing thoughts and feelings related to pain experiences. Responses are scored on a 5-point Likert scale ranging from 0 (“not at all”) to 4 (“all the time”), with the total score calculated by summing all item scores. The PCS features 3 subscales: rumination, magnification, and helplessness. The instrument has been translated, culturally adapted, and validated in Greek. Written permission for its use was obtained from MAPI Research Trust, which holds the intellectual property rights [26]. The authorized Greek version was provided for use in this study. The PCS was selected because it is one of the most widely used instruments for assessing pain catastrophizing in both clinical and research settings. In addition, its relatively short format facilitates its administration alongside other study questionnaires.

Pain intensity is measured using the Visual Analog Scale (VAS) immediately after the mammography examination. The VAS features a horizontal 10-cm line labeled with “no pain” (0) and “worst imaginable pain” (10). Participants mark the point on the line that best represents their subjective pain. The final pain score is determined by measuring the distance in centimeters from the left endpoint to the marked point. The VAS is a reliable and widely accepted tool for quantitative pain assessment in both clinical and research settings [27].

In addition to the psychometric instruments, participants are asked to answer 3 open-ended questions (Table 1) to qualitatively explore their mammography experiences:

1. Please describe your overall experience with the mammography examination you just had in your own words (you can mention anything that stood out to you, whether positive or negative).
2. Which factors do you believe most influenced your experience during mammography? (For example, these may relate to the procedure, equipment, staff, or your personal characteristics.)
3. What do you think could make the mammography experience less painful or more comfortable for you and other women in the future?

**Table 1.** Summary of study variables, measurement instruments, and assessment time points.

Variable	Instrument	Assessment time point
Demographic and clinical characteristics	Demographic and Clinical Information Form	Before mammography
Anxiety and depression	PHQ-4^a^	Before mammography
Pain catastrophizing	PCS^b^	Before mammography
Personality traits	TIPI^c^	Before mammography
Pain intensity	VAS^d^	Immediately after mammography
Mammography experience	Three open-ended questions	Immediately after mammography

a PHQ-4: Patient Health Questionnaire-4.

b PCS: Pain Catastrophizing Scale.

c TIPI: Ten-Item Personality Inventory.

d VAS: Visual Analog Scale.

Responses to the open-ended questions are documented in writing by the participants themselves, with no limit on length. Psychological variables are evaluated before mammography, while pain perception is measured within 5 minutes of completing the examination. All questionnaires are completed in a designated waiting area under the supervision of the principal investigator. Given the uniform nature of the sample (women undergoing routine screening mammography), the same tools and procedures are consistently used for all participants. In any subgroup analyses (such as first-time vs repeat mammography), the measurement process remains the same. Finally, a pilot study was conducted with a small sample (n=10) to assess the clarity of the questions and the practicality of the data collection process.

### Bias

The study aims to minimize potential sources of systematic bias that could impact the reliability and internal validity of the results. Preventive measures are implemented throughout the study design, data collection, and data analysis stages.

#### Selection Bias

To minimize the risk of selection bias, the following measures are implemented:

- A total (consecutive) sampling method is used, including all women undergoing routine screening mammography who meet the predefined inclusion criteria throughout the entire study period. No prior sample selection is performed to improve representativeness.
- Participation is entirely voluntary. Before enrolling, all eligible women receive a detailed Participant Information Sheet and provide written informed consent, ensuring that no coercion, obligation, or institutional pressure is present.
- Data collection takes place every day of the week and during all available appointment times (morning and afternoon sessions). This approach is expected to enhance the representativeness of the study sample and reduce, but not eliminate, the risk of selection bias associated with recruitment during limited days or time periods.
- No further exclusion criteria beyond those already set are used, reducing the risk of selective inclusion.

#### Information Bias

To minimize the risk of information bias, the following measures are implemented:

- All data are collected through anonymous self-report surveys, with no personal identifying information recorded.
- Questionnaires are completed in a private, quiet waiting area without the presence of third parties or health care staff to ensure honest and free responses.
- Standardized instructions are provided to all participants, minimizing the researcher’s subjective influence and decreasing observer bias.
- The questionnaire is administered in the same order for all participants to prevent order effects.

#### Measurement Bias

The following measures are used to minimize the risk of measurement bias:

- Validated and standardized psychometric tools with proven reliability and validity in the Greek population will be used.
- Data are collected by the same researcher under identical conditions for all participants.
- Mammography examinations are conducted by the same radiologic technologist, using consistent equipment and a standardized compression protocol to reduce variability in the pain stimulus.
- Questionnaire administration and data collection take place at specific time points before and after the examination, preventing potential bias caused by timing or delay effects.

#### Recall Bias

To reduce the risk of recall bias, the following measures are applied:

- Participants complete the questionnaires immediately before the mammography examination and within a few minutes after it, without needing to recall past experiences.
- The questions focus on current emotional states and immediate pain perception, thereby lowering the risk of inaccurate responses caused by time lag.

### Study Size

The sample size was determined by the planned primary statistical analysis, namely hierarchical multiple linear regression. A formal power analysis was conducted using G*Power (version 3.1) for linear multiple regression: fixed model, *R*² deviation from zero. The model included 17 predictors: anxiety, depression, pain catastrophizing, 5 broad personality traits, 8 a priori selected confounding variables, and 1 interaction term between anxiety and previous mammography experience. Assuming a medium effect size (*f*²=0.15), selected in accordance with Cohen recommendations for multiple linear regression, an α level of .05, statistical power of 0.90, and 17 predictors, the minimum required sample size was approximately 179 participants [28].

The planned sample size of 380 women therefore exceeds the minimum required sample size and provides approximately 22 participants per predictor, reducing the risk of overfitting. Across the 2 participating screening centers, approximately 1000 women undergo screening mammography annually. Therefore, recruiting 380 participants is considered feasible within the study period.

### Quantitative Variables

All quantitative variables (VAS, PHQ-4, PCS, and TIPI scores) will be analyzed as continuous variables to maximize statistical power. The normality of the distributions and the linearity of the associations will be checked before conducting inferential analyses. For descriptive purposes, selected variables will also be reported using clinically established categories (eg, mild, moderate, and severe pain for VAS scores).

### Ethical Considerations

This study is conducted in accordance with the core principles of research ethics and integrity as outlined in the Declaration of Helsinki, the guidelines of the International Committee of Medical Journal Editors, and the General Data Protection Regulation (European Union Regulation (EU) 2016/679).

Ethics approval was granted by the Cyprus National Bioethics Committee on March 4, 2026 (Reference: EEBK EP 2026.01.86). Institutional permission was also obtained from the participating private radiology center and from the Ministry of Health of the Republic of Cyprus for data collection in the public health care setting. In addition, written permission to use all psychometric scales and questionnaires was obtained from the original authors or copyright holders, as required. Throughout all stages of the study, full confidentiality and anonymity of the data are maintained. Each participant is assigned a unique identification code, which is used on all data collection forms. Personal identifying information, such as names or other direct identifiers, is not recorded, reported, or disclosed. Before completing the questionnaires, participants receive detailed oral and written information about the study’s purpose, content, procedures, and voluntary nature. Sufficient time is provided for questions and clarifications, and written informed consent is obtained. Participants are informed that they may withdraw from the study at any time without consequences.

Data are accessible only to the research team and are used exclusively for the purposes of this study. All paper-based data collection and coding forms are securely stored in a locked drawer in the principal investigator’s office for 36 months after completion of the study. After this period, the data will be securely shredded. Collectively, these measures ensure participant anonymity and data confidentiality, and access is restricted solely to the research team.

Participants do not receive any financial compensation or incentives for participating in this study.

### Statistical Methods

Data will be analyzed using descriptive and inferential statistics to address the study objectives and hypotheses. Categorical variables (eg, marital status, employment status, menopausal status) will be presented as absolute and relative frequencies. Continuous variables (eg, age, VAS, PHQ-4, PCS, and TIPI scores) will be summarized with means and SDs, or, when distributions are nonnormal, with medians and IQRs. Normality will be assessed using the Kolmogorov-Smirnov test and visual inspection of histograms and Q-Q plots. Bivariate analyses will use Pearson or Spearman correlation coefficients, depending on the data distribution. Group comparisons (eg, first-time vs repeat mammography) will be conducted with independent sample *t* tests or Mann-Whitney *U* tests, as appropriate. To examine the independent relationship between psychological factors and pain intensity, hierarchical multiple linear regression will be used. In the first step, demographic and clinical confounders will be included; in the second step, psychological variables (anxiety, depression, and pain catastrophizing) will be included; and in the third step, personality characteristics will be included. Multicollinearity will be assessed using the variance inflation factor. The assumptions of linear regression will be evaluated by examining the normality of residuals, homoscedasticity using residual plots, and the independence of residuals using the Durbin-Watson statistic. The internal consistency of the PHQ-4 and the PCS will be assessed using Cronbach α. Given the 2-item structure of each TIPI personality dimension, internal consistency coefficients will be interpreted cautiously, consistent with the instrument developers’ recommendations.

Statistical significance in the final model will be set at *P*<.05 (2-tailed). Results will be reported with adjusted regression coefficients (adjusted B and standardized β) and their 95% CIs. As a secondary analysis, previous mammography experience (first-time vs repeat examination) will be tested as a potential moderator of the relationship between anxiety and pain perception using interaction terms and stratified analyses. Bonferroni correction will not be applied to the primary multivariable regression analyses because these analyses are based on prespecified hypotheses and a predefined conceptual framework rather than exploratory hypothesis testing. Adjustment for multiple comparisons will be considered only for exploratory secondary pairwise analyses, where appropriate. For missing data below 5%, listwise deletion will be used; for missing data between 5% and 10%, multiple imputation will be applied, with comparisons to complete-case analyses. Sensitivity analyses will be performed using nonparametric methods and by excluding extreme values (>3 SD). Statistical analyses will be carried out using IBM SPSS Statistics version 28.0 (or later).

The planned statistical analyses were selected to directly address the study objectives and hypotheses. Correlation analyses and hierarchical multiple linear regression will be used to examine the association between anxiety and pain intensity while adjusting for potential demographic and clinical confounders. Group comparisons will assess pain intensity between women undergoing their first mammography and those with prior mammography experience. Hierarchical regression analyses will also evaluate the independent contribution of depression, pain catastrophizing, and personality traits to pain perception after adjusting for potential confounding variables.

Responses to the 3 open-ended questions will be analyzed using reflexive thematic analysis, following the approach proposed by Braun and Clarke [29] and guided by their updated recommendations for good practice [30]. The analysis will follow the 6 phases: familiarization with the data, generation of initial codes, development of candidate themes, review and refinement of themes, definition and naming of themes, and production of the final report [29]. Two members of the research team will engage in ongoing reflexive discussions throughout the coding and theme development process to critically reflect on emerging interpretations and enhance the transparency and rigor of the analysis, consistent with the principles of reflexive thematic analysis [28]. Reflexivity will be maintained through ongoing critical reflection on the researchers’ perspectives, assumptions, and potential influence on data interpretation [28]. An audit trail documenting coding decisions, theme development, and analytical decisions will be maintained throughout the study to enhance methodological transparency and dependability. Credibility will be strengthened through regular discussions between the researchers during data interpretation, while confirmability will be supported through reflexive documentation to ensure that the findings remain grounded in participants’ responses. Transferability will be facilitated by providing a rich description of the study context, participant characteristics, and analytical procedures, enabling readers to assess the applicability of the findings to similar settings. The qualitative findings will be interpreted alongside the quantitative results to provide a more comprehensive understanding of women’s experiences during mammography and to contextualize the quantitative findings.

## Results

Ethics approval was granted by the Cyprus National Bioethics Committee (Reference: EEBK EP 2026.01.86) on March 4, 2026. Institutional permissions were also obtained from the participating private radiology center and from the Ministry of Health of Cyprus for data collection in the public health care setting. Participant recruitment and data collection began in March 2026. By June 2026, 50 women meeting the eligibility criteria had been enrolled in the study. Recruitment and data collection are expected to continue until September 2026. Data analysis is anticipated to be completed by December 2026, with dissemination of findings through conference presentations and the submission of manuscripts to peer-reviewed journals planned for 2027. As this is a study protocol and participant recruitment is ongoing, no analytical results are currently available. Following study initiation, the study setting was expanded to include an additional public radiology center in Cyprus to facilitate participant recruitment and enhance the representativeness of the study sample. No other substantial changes to the study protocol have been made.

## Discussion

### Principal Findings

This study aims to investigate the association between psychological factors, personality traits, and pain perception during routine screening mammography. Building on prior literature, the study examines whether psychological factors assessed before the examination—specifically pain catastrophizing, anxiety, depressive symptoms, and personality traits—are associated with perceived pain after mammography, as measured by the Visual Analog Scale. In addition, demographic and clinical factors, such as age, prior mammography experience, and other relevant characteristics, are also evaluated for potential associations with reported pain intensity. Overall, this study seeks to further explore the role of women’s psychological state prior to mammography as a potential determinant of pain experience and, if supported by the findings, to contribute to the development of targeted, patient-centered interventions to improve comfort and adherence to breast cancer screening programs.

### Comparison With Available Literature

Previous studies have demonstrated that pain experienced during mammography is influenced not only by technical factors but also by psychological variables such as anxiety, fear, and pain catastrophizing. Several investigations have reported positive associations between higher pre-examination anxiety or catastrophizing and increased pain perception during mammography. However, findings across studies remain inconsistent, with some reporting weak or nonsignificant associations, potentially due to differences in study design, populations, and measurement tools [13,14].

Furthermore, our previously published systematic review on this topic identified important evidence gaps, including inconsistent evidence regarding depressive symptoms, pain catastrophizing, and personality traits, as well as a lack of studies simultaneously examining multiple psychological factors within the same analytical framework [20]. Building on the evidence gaps identified in the systematic review, this cross-sectional study adopts a comprehensive approach by concurrently assessing anxiety, depressive symptoms, pain catastrophizing, and personality traits in women undergoing routine screening mammography in clinical practice. By addressing these gaps, this study extends previous evidence and advances understanding of the psychological determinants of mammography-related pain, with potential implications for the development of targeted, patient-centered interventions.

### Study Limitations

This study has several limitations to consider when interpreting the results. First, its cross-sectional design does not allow for causal relationships between psychological factors and pain perception to be established—only associations—and reverse causality cannot be excluded. Although valid and reliable psychometric tools are used, data collected via self-report questionnaires may be affected by response bias, including social desirability bias, subjective interpretation, and inaccuracies in reporting psychological symptoms. Additionally, the use of brief assessment instruments such as the PHQ-4 and TIPI, although practical for minimizing participant burden, may provide a less comprehensive evaluation of psychological factors and personality traits compared with more extensive psychometric measures. Another limitation is that the study does not directly assess pain expression styles, emotional suppression, or health care professionals’ responses to pain communication, which may influence both the subjective experience of pain and the way pain is reported during mammography. Because data collection had already commenced, these factors could not be incorporated into this study design and should be considered in future research. Lastly, requiring participants to be proficient in Greek (reading, writing, and comprehension) excludes women who do not meet this criterion, which could impact the external validity of the study.

### Broader Implications and Conclusions

Understanding the role of psychological factors and personality traits in pain perception during mammography has significant clinical and public health implications. If psychological variables such as anxiety, depressive symptoms, pain catastrophizing, and specific personality characteristics are confirmed as key determinants of pain experience, early identification of women at higher risk could allow for the implementation of targeted, patient-centered interventions before the examination. Such interventions might include improved communication, psychological support, and personalized preparatory strategies to reduce distress and increase comfort during mammography.

On a broader level, if supported by the study findings, incorporating the evaluation of psychological factors and personality traits into routine breast imaging may enhance women’s experiences during mammography and potentially improve participation in breast cancer screening programs. If supported by the study findings, this research could contribute to evidence-based, holistic approaches in breast imaging and highlight the potential value of integrating psychological and personality assessments into screening environments.

### Dissemination of the Study Results

Upon completion of the study, the findings will be disseminated through publications in peer-reviewed scientific journals and presentations at national and international conferences in the fields of radiology, public health, and behavioral medicine. Additionally, the results will be communicated to health care professionals involved in breast imaging to facilitate their implementation in clinical practice. All dissemination efforts will be conducted in a manner that fully safeguards participant confidentiality and anonymity.

## Supplementary material

10.2196/92804 Multimedia Appendix 1 Demographic and clinical information.

10.2196/92804 Checklist 1 STROBE checklist.

## Abbreviations

PCS: Pain Catastrophizing Scale
PHQ-4: Patient Health Questionnaire-4
STROBE: Strengthening the Reporting of Observational Studies in Epidemiology
TIPI: Ten-Item Personality Inventory
VAS: Visual Analogue Scale
