# Supplementary material for: Psychological Factors and Personality Traits Influencing the Experience and Perception of Pain in Women Undergoing Mammography: Protocol for a Cross-Sectional Study
Source: JMIR Res Protoc. 2026 Aug 25;15:e92804. doi: 10.2196/92804 (PMC13505197; doi:10.2196/92804)
Supplement: Supplementary file 1 [file resprot-v15-e92804-s001.pdf]

Appendix 1. Demographic and Clinical Information Form

The following questionnaire was completed by all participants prior to undergoing mammography.

Part I. Demographic and Clinical Characteristics

Please answer the following questions. All information will remain confidential and will be used exclusively for research purposes.

A. Demographic Information

1. What is your age?

☐ ____ years

2. What is the highest level of education you have completed?

☐ Primary education

☐ Lower secondary education

☐ Upper secondary education

☐ Postsecondary non-tertiary / Technical education

☐ Bachelor's degree

☐ Master's degree

☐ Doctoral degree

3. Which educational system did you primarily attend?

☐ Greek

☐ Cypriot

☐ Other: __________

4. What is your marital status?

☐ Single

☐ Married / Cohabiting

☐ Divorced

☐ Widowed

☐ Other: __________

5. Do you have children?

☐ Yes → Number of children: ____

☐ No

6. Where do you permanently reside?

☐ Urban area

☐ Semi-urban area

☐ Rural area

City/Region: __________

7. Who do you currently live with?

☐ Alone

☐ With family

☐ With partner

☐ With children

☐ Other: __________

8. What is your current employment status?

☐ Full-time employed

☐ Part-time employed

☐ Unemployed

☐ Retired

☐ Homemaker

☐ Other: __________

9. How would you describe your financial status? (Self-reported)

☐ Low

☐ Moderate

☐ Good

☐ Very good

B. Health Status and Clinical Characteristics

10. How would you rate your overall health?

☐ Excellent

☐ Very good

☐ Good

☐ Fair

☐ Poor

11. How would you rate your psychological well-being?

☐ Very good

☐ Good

☐ Fair

☐ Poor

☐ Very poor

12. Do you currently smoke?

☐ Yes

☐ No

☐ Former smoker

13. Have you been diagnosed with any chronic disease?

☐ No

☐ Cardiovascular disease

☐ Diabetes mellitus

☐ Thyroid disorder

☐ Musculoskeletal disorder

☐ Other: __________

14. Have you been diagnosed with a neurological disorder (e.g., Alzheimer's disease, dementia, traumatic brain injury)?

☐ Yes

☐ No

If yes, please specify: __________

15. Have you been diagnosed with a serious physical illness (e.g., cancer, including breast cancer)?

☐ Yes

☐ No

If yes, please specify: __________

16. Have you been diagnosed with a severe psychiatric disorder (e.g., schizophrenia, bipolar disorder, psychosis)?

☐ Yes

☐ No

If yes, please specify: __________

C. Hormonal and Gynaecological History

17. Are you currently pregnant?

☐ Yes

☐ No

18. Are you currently breastfeeding?

☐ Yes

☐ No

If yes, total duration of breastfeeding: ____ months

19. Have you reached menopause?

☐ Yes

☐ No

☐ Perimenopausal

20. (If not menopausal)

Your menstrual cycle is:

☐ Regular

☐ Irregular

21. How many childbirths have you had?

☐ 0

☐ 1

☐ 2

☐ 3

☐ 4 or more

D. Breast and Mammography History

22. Have you noticed any clinical signs suggestive of breast pathology (e.g., palpable lump, nipple changes)?

☐ Yes

☐ No

If yes, please specify: __________

23. Do you have breast implants?

☐ Yes

☐ No

24. What is your usual bra cup size?

☐ A

☐ B

☐ C

☐ D

☐ DD/E

☐ F or larger

25. Have you previously undergone mammography?

☐ No, this is my first mammogram

☐ Yes, I have undergone mammography before

26. When was your most recent mammogram?

☐ I have never had a previous mammogram

☐ Year: ______

27. How would you describe the pain you experienced during your previous mammogram?

☐ No pain

☐ Mild pain

☐ Moderate pain

☐ Severe pain

☐ Very severe pain
