# Supplementary material for: Psychological Factors and Personality Traits Influencing the Experience and Perception of Pain in Women Undergoing Mammography: Protocol for a Cross-Sectional Study
Source: JMIR Res Protoc. 2026 Aug 25;15:e92804. doi: 10.2196/92804 (PMC13505197; doi:10.2196/92804)
Supplement: Supplementary file 2 [file resprot-v15-e92804-s002.pdf]

**STROBE Checklist (Cross-sectional Study Protocol)**

| | | |
|---|---|---|
| 1.Title and Abstract | (a) Indicate the study design using a commonly accepted term in the title or abstract.<br>(b) Provide in the abstract an informative summary of the study rationale, objectives, methods, and expected contribution. | Pages 1-2 |
| **Introduction**<br>2. Background | Explain the scientific background and rationale for the study. | Pages 2-4 |
| 3. Objectives | State the specific objectives and prespecified hypotheses | Pages 4-5 |
| **Methods**<br>4. Study design | Present the key elements of the study design early in the manuscript. | Page 5 |
| 5.Setting | Describe the setting, locations, and relevant dates, including recruitment and data collection period. | Pages 5- 6 |
| 6.Participants | Provide eligibility criteria and describe the source and methods of participant selection. | Page 6 |
| 7.Variables | Clearly define the primary outcome, exposures, predictors, potential confounders, and effect modifiers. | Pages 6- 7 |
| 8. Data sources/measurement | Describe the data sources and methods of measurement for each variable. | Pages 7-9 |
| 9.Bias | Describe potential sources of bias and strategies used to minimize them. | Pages 9-10 |
| 10. Study size | Explain how the sample size was determined. | Page 10 |

| | | |
|---|---|---|
| 11. Quantitative variables | Explain how quantitative variables will be handled in the planned analyses. | Page 10 |
| 12. Statistical methods | (a) Describe all planned statistical analyses, including methods for controlling confounding.<br>(b) Describe planned subgroup and interaction analyses.<br>(c) Explain how missing data will be handled.<br>(d) Describe any analytical methods relevant to the sampling strategy, if applicable.<br>(e) Describe planned sensitivity analyses. | Page 11 |
| **13.Results (Protocol-specific adaptation)** | Report the current recruitment status and, if applicable, the number of participants enrolled to date. | Page 12 |
| **14. Discussion (Protocol-specific adaptation)** | Key results<br>Summarize the expected contribution of the study in relation to the objectives.<br><br>Limitations<br>Discuss anticipated methodological limitations and possible sources of bias.<br><br>Interpretation<br>Provide a cautious interpretation of the study rationale, considering limitations and relevant evidence.<br><br>Generalisability<br>Discuss the potential | Pages 12-15 |

| | | |
|---|---|---|
| | generalisability of the future findings. | |
| **Other Information** | Funding<br>State the funding source and the role of the funders.<br><br>Ethics<br>Report ethical approval and informed consent procedures. | Page 15<br><br><br>Pages 10-11 |
